# A new and general approach to signal denoising and eye movement classification based on segmented linear regression

**DOI:** 10.1038/s41598-017-17983-x

**Published:** 2017-12-18

**Authors:** Jami Pekkanen, Otto Lappi

**Affiliations:** University of Helsinki, Cognitive Science, Helsinki, 00014 Finland

## Abstract

We introduce a conceptually novel method for eye-movement signal analysis. The method is general in that it does not place severe restrictions on sampling frequency, measurement noise or subject behavior. Event identification is based on segmentation that simultaneously denoises the signal and determines event boundaries. The full gaze position time-series is segmented into an approximately optimal piecewise linear function in *O*(*n*) time. Gaze feature parameters for classification into fixations, saccades, smooth pursuits and post-saccadic oscillations are derived from human labeling in a data-driven manner. The range of oculomotor events identified and the powerful denoising performance make the method useable for both low-noise controlled laboratory settings and high-noise complex field experiments. This is desirable for harmonizing the gaze behavior (in the wild) and oculomotor event identification (in the laboratory) approaches to eye movement behavior. Denoising and classification performance are assessed using multiple datasets. Full open source implementation is included.

## Introduction

Humans and other foveate animals – such as monkeys and birds of prey – visually scan scenes with a characteristic fixate-saccade-fixate pattern: periods of relative stability are interspersed with rapid shifts of gaze. During “fixation” the visual axis (and high-resolution foveola) is directed to an object or location of interest. For humans, the duration of the periods of stability is on the order of 0.2–0.3 s, depending on a number of factors such as task and stimulus complexity. The typical duration of saccadic eye movements in on the order of 0.01–0.1 s, depending systematically on the amplitude of the movement^1^.

If the scene contains moving target objects, or when the observer is moving through it, then stabilization of gaze on a focal object or location requires a “tracking fixation”, i.e. a smooth pursuit eye movement. Here the eye rotates to keep gaze fixed on the target. Also, when the observer’s head is bouncing due to locomotion or external perturbations, gaze stabilization involves vestibulo-ocular and optokinetic compensatory eye movements. In natural behavior, all the eye movement “types” mentioned above are usually simultaneously present, and cannot necessarily be differentiated from one another in terms of oculomotor properties or underlying neurophysiology^2–5^.

It is possible to more or less clearly experimentally isolate each of the aforementioned “types” in experiments that tightly physically constrain the visible stimuli and the patterns of movement the subject is allowed to make. Much of what we know about oculomotor control circuits is based on such laboratory experiments where the participant’s head is fixed with a chin rest or a bite bar, and the stimulus and task are restricted so as to elicit only a specific eye movement type. In order to understand how gaze control is used in natural behavior, however, it is essential to be able to meaningfully compare oculomotor behavior observed in constrained laboratory recordings to gaze recordings “in the wild”^2,5–7^.

Laboratory grade systems typically have very high accuracy and very low noise levels. Sampling frequencies may range from 500 to as high as 2000 Hz. As the subject’s behavior is restricted, it is possible to tailor custom event identification methods that rely on only the eye movement type of interest being present in the data (and would produce spurious results with data from free eye movement behavior). On the other hand, mobile measuring equipment has much lower accuracy and relatively high levels of noise, with sampling frequency typically between 30 and 120 Hz. The subject’s behavior is complex, calling for robust event identification that works when all eye movement types are simultaneously present. Unfortunately, these different requirements have led, and increasingly threaten to lead, the methodologies and concepts of “laboratory” and “naturalistic” research into diverging directions. For wider generalizability of results, it would be desirable to analyze eye movement events in a similar way across task settings, by using event detection methods that do not rely on restrictions or assumptions which are not valid for most natural behavior.

Here, we introduce *Naive Segmented Linear Regression* (NSLR), a new method for eye-movement signal denoising and segmentation, and a related event classification method based on Hidden Markov Models (NSLR-HMM). The approach is *novel* in that it differs in concept from the traditional workflow of pre-filtering, event detection and segmentation. Instead, it integrates denoising into segmentation which is now the first – rather than the last – step in the analysis, and then performs classification on the denoised segments (rather than sample-to-sample). The method is *general* in two ways: Firstly, it performs a four-way identification of fixations, saccades, smooth pursuits and post-saccadic oscillations, which allows for experiments with complex gaze behavior. Secondly, it can be directly applied to noisy data to recover robust gaze position and velocity estimates, which means it can be used on both high-quality lab data and more challenging mobile data on natural gaze behavior. The method also automatically estimates the signal’s noise level and determines gaze feature parameters from human classification examples in a data-driven manner, requiring minimal manual parameter setting.

We believe this is an important development direction for eye movement signal analysis as this can help counteract the historical tendency in the field of eye tracking to develop operational definitions of eye movement “types” that are based on very specific and restrictive oculomotor tasks and event identification methods tailor-made for them (and then “reify” the types as separate phenomena). In contrast, our method has a number of desirable features that compare favorably with the state of the art and will in part help harmonize the traditional oculomotor and more naturalistic gaze behavior research traditions:

1. The NSLR method is based on a few simple and intuitively transparent basic concepts.
2. It requires no signal preprocessing (e.g. filtering, as denoising is inherent into the segmentation step), and no user-defined filtering parameters.
3. Segmentation is conceptually parsimonious and uses only a few parameters (that can be estimated from the data itself).
4. No “ground truth” training data from human annotators is necessary for segmentation. (Human coding data is needed for classification, which is treated as a separate subproblem).
5. The HMM classifier can identify four types of eye movement (saccade, PSO, fixation, pursuit).
6. This classification uses global signal information. (It is not based on sample-wise application simple criteria such as duration or velocity thresholds).
7. Because of its wide range of oculomotor event identification and powerful denoising performance it can be used for both low-noise laboratory data in tasks that only elicit one or two types of oculomotor events and high-noise field data collected during complex behavior. This is desirable for harmonizing the gaze behavior (in the wild) and oculomotor event identification (in the laboratory) perspectives on eye movement behavior.

Full C++ and Python implementation of the method is available under an open source license at https://gitlab.com/nslr/.

## Gaze Signal Denoising

All eye-movement recordings contain some level of noise, ie high frequency variation in the signal not caused by movement of the eye itself. Total noise is a combination of several noise mechanisms, but mainly due to the vibration of the recording equipment in relation to the eyes with some additional noise inherent in the recording method (e.g. image sensor noise in optical tracking and environment’s electromagnetic noise in electro-oculography or scleral search coil recording). The noise level can vary by multiple orders of magnitude depending on the environment and equipment, from around 0.01° with scleral search coils or high-quality optical equipment in laboratory conditions, to around 0.3° with head-mounted cameras in mobile settings^8^, to well over 1° in mobile recording with remote cameras^9^.

The ramifications of the measurement noise on event identification and on the interpretation of the results depend on how the gaze signal is analyzed, but in general, the noise introduces two kinds of challenges. Firstly, measurement noise directly leads to noise in estimates of the gaze features, such as gaze position or gaze angular velocity. Secondly, it causes problems when classifying the different eye movement types by using these gaze signal features, especially velocity.

Most contemporary event identification algorithms use sample-level features, e.g. sample-to-sample velocities^10–12^ which are especially prone to be affected by high-frequency noise. In order to avoid erroneous detections due to the noise, most of the algorithms rely on some kind of signal denoising, particularly in applications where signals need to be recorded in more challenging environments such as driving, outdoors, infant or nonhuman data, or free eye-hand coordination (cf.^13,14^).

A common denoising approach is to use some kind of linear time-invariant (LTI) filter, such as the Butterworth, the Gaussian or the Savitzky–Golay filter. These techniques have some favorable properties: they are well understood, high-quality implementations are readily available, they have low computational requirements and many can be run in online mode. However, especially in high-noise scenarios, the low-pass (noise smoothing) part of the filters can significantly distort the relevant signal features, such as saccade velocities. This can be somewhat remedied by adding a high-pass (sharpening) component, but this can introduce artifacts of its own, especially it may introduce false oscillations (“ringing”). In general, finding an LTI parameterization that balances the noise reduction and signal distortion is a somewhat challenging problem^15^.

Some of the LTI filters’ issues may be ameliorated with non-LTI filtering. Some such filters used for gaze signals filters include the median filter and its adaptations^16^, the Kalman filter^17^ and the Bilateral filter^11^. Although these do help with some of the LTI filters’ problems, they still share a main characteristic: the resulting signal is some sort of aggregate of a neighborhood of samples at any given point. Importantly for gaze signals, this means that every denoised sample is at least somewhat dependent on its full neighborhood, e.g. location of the preceding fixation’s samples has an effect on the denoised samples of the next fixation, although in reality, such dependency is largely eliminated by the saccade between the fixations.

On the other hand, the usually relatively small neighborhood limits the denoising algorithms’ accuracy, as they can’t use the full data. Methods for finding an estimate of the global optimum under some assumptions do exist, but most of these, such as sequential Bayesian smoothing and smoothing splines^18^, have prohibitive computational complexity and/or assume a signal form that is a poor fit for eye-movement data. One possible exception is the Total Variation filter and related techniques^19^, which tend to form stepwise constant estimates and can be approximated using fast iterative methods^20^, although to our knowledge these have not been used for gaze signal denoising.

### Denoising by Naive Segmented Linear Regression

Our proposed method is based on the assumption that in most situations the gaze position signal is well approximated by a piecewise linear function, and events correspond to the pieces of such function. The method finds a piecewise linear function that approximately minimizes the approximation error, while taking account prior knowledge of typical eye movement characteristics.

A similar problem is often discussed as *changepoint detection*, and it’s been that a globally optimal solution for such problems can be found in linear time using the *Pruned Exact Linear Time* (PELT) method^21^. The PELT method builds on a previous idea known as the *Optimal Partitioning* method^22^. Under rather realistic assumptions, for each measurement of the time-series, we can form two hypotheses: either the current segment continues or a new one starts. Furthermore, if the measurement noise and probability of a new segment starting are assumed to be independent of the segmentation, a new segment starting from the currently most likely past segmentation has always the highest total likelihood. This result can be quite straightforwardly used to come up with an algorithm that finds the optimal segmentation, but with *O*(*n* ^2^) computational complexity^22^, which renders it impractical for long and high sampling rate series such as produced by eye trackers.

The PELT method reduces the method’s complexity to *O*(*n*) by *pruning* segmentation hypotheses that can never yield the optimal result. Briefly, as starting a new segment always gives a lower regression error, segmentation hypotheses with a smaller likelihood than the newly formed new segment hypothesis can never reach the new hypothesis’ likelihood, and thus can be ignored in future calculations.

The discussed changepoint detection methods are usually applied to piecewise constant functions, but we utilize the PELT method to find an approximation of maximum likelihood piecewise linear segmentation assuming Gaussian distributed measurement noise in *O*(*n*) time. A central requirement for most dynamic programming methods, such as PELT, is that the problem has *optimal substructure*, i.e. it can be decomposed into independent subproblems. In our case of finding a maximum likelihood estimate, this means that the likelihood function must have a form where its value at a given step doesn’t depend on the future steps. The likelihood function of a continuous piecewise regression clearly doesn’t fulfill this requirement; changes anywhere in the function affect all of the segments and thus the likelihood.

In order to satisfy the optimal substructure criterion, we approximate the maximum likelihood segmentation by greedily satisfying the continuity condition, ie force each new linear segment to start with the currently predicted value of the previous segment. For the final estimate of the regression, we fit an optimal continuous regression to the segmentation obtained using the greedy continuity. Due to the greediness, we refer to the method as Naive Segmented Linear Regression (NSLR). The method is presented more formally along with a Python implementation in a Supplementary Note. More performant C++ implementation with Python bindings is available at http://gitlab.com/nslr/nslr.

In order to avoid the trivial solution of every sample starting a new segment, segmented regression requires additional regularization to “penalize” too short segments. A common approach is to use a generic information criterion that penalizes for model complexity^21^, however, these are based on asymptotic behavior of certain models – such as ARMA models and ordinary linear regression – which likely differ from our model regarding model complexity and overfitting^23^. Furthermore, incorporating knowledge of the underlying signal into the penalization should improve the denoising performance. In order to take account specifics of our model and characteristics of gaze signals, we present numerically estimated penalty factors based on simulated eye movement data.

#### Measurement noise and segmentation penalty parameters

The final objective function depends only on two parameters: variance of the measurement noise and the penalty factor for starting a new segment. However, for eye movement analysis especially the penalty factor is rather far removed from how practitioners assess the signal properties and cannot be easily expressed as a verbal rule of thumb or intuited from the raw data; as indicated by our simulation results, an optimal penalization parameter value depends on multiple characteristics of the underlying signal. For a more intuitive parameterization, we use synthetic eye movement data simulating different eye movement scenarios and formulate the penalty factor as a function of saccade amplitudes, slow eye movement durations, and slow eye movement velocities.

Given a penalty factor, the measurement noise level can be optimized automatically. This is done iteratively by observing standard deviation between reconstruction and the signal and using this as the noise estimate for the next iteration. The iteration is stopped when a standard deviation observed earlier is repeated. The standard deviation of the signal itself is used as the initial estimate.

The automated estimate can mostly account for uncorrelated high-frequency noise, but gaze signals also do have some correlated noise (see Human eye movement data) and minute eye movements (e.g. microsaccades or tremors) that are generally considered irrelevant for macro level oculomotor event identification. In order to treat these as “noise”, we propose adding an additional “structural error” value to the estimated noise, with value typically around 0.1°, especially when using the segmentation for event identification with low noise signals.

Combined with the typical eye movement characteristics, provided here with the algorithm, the automatic noise estimating means that the denoising and segmentation method can be in most cases used without any user specified parameters. The parameter estimation method and resulting parameterizations are presented in detail in the Supplementary Methods.

### Denoising performance results

We study the denoising performance of the NSLR method by measuring its ability to recover the recorded eye position from a noisy signal. The performance is compared to the Wiener filter, which, as the optimal linear time-invariant filter for this measure provides an upper bound of performance for any LTI filter, and to the nonlinear Total Variation denoising filter^19^. Total Variation filter was chosen as the reference nonlinear filter based on some favorable theoretical properties (see Gaze Signal Denoising) and on informal experimentation where it compared favorably with various nonlinear filters. The parameters of both reference methods are optimized against the ground truth data (see Denoising benchmarking), thus providing approximate upper bound of their performance. Note that NSLR uses the default parameterization and automatically estimates the noise level, without access to the ground truth data (see Measurement noise and segmentation penalty parameters).

**Figure 1:**
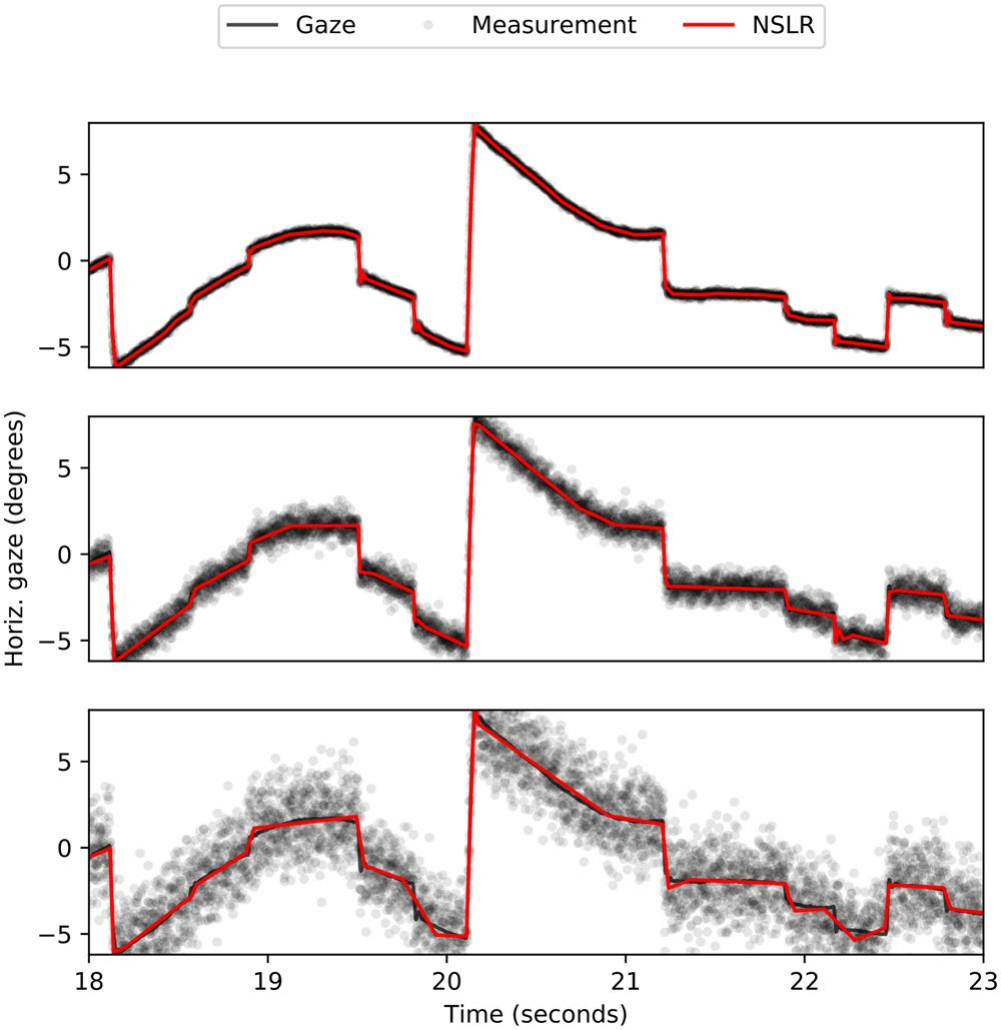
Horizontal gaze position time series excerpt of a recording where the participant is watching a movie clip^24^. The panels show three different levels of simulated measurement noise (standard deviation from top to bottom: 0.1°, 0.5°, 1.5°). Red lines indicate the denoising result of NSLR (the identified segments) with the default parameters and automatically-from-data inferred noise level. For both axes, see Supplementary Figures S1–S3.

We use two elsewhere recorded high-quality gaze signal datasets which include both stationary and moving targets (see Human eye movement data), and one simulated dataset (see Simulated eye movement data) as recordings. Recording noise is simulated by additive axis-independent Gaussian noise with the standard deviation ranging from 0.03° to 3.0°. An excerpt from a recording from^24^ with NSLR denoising at different noise levels is plotted in Fig. 1.

**Figure 2:**
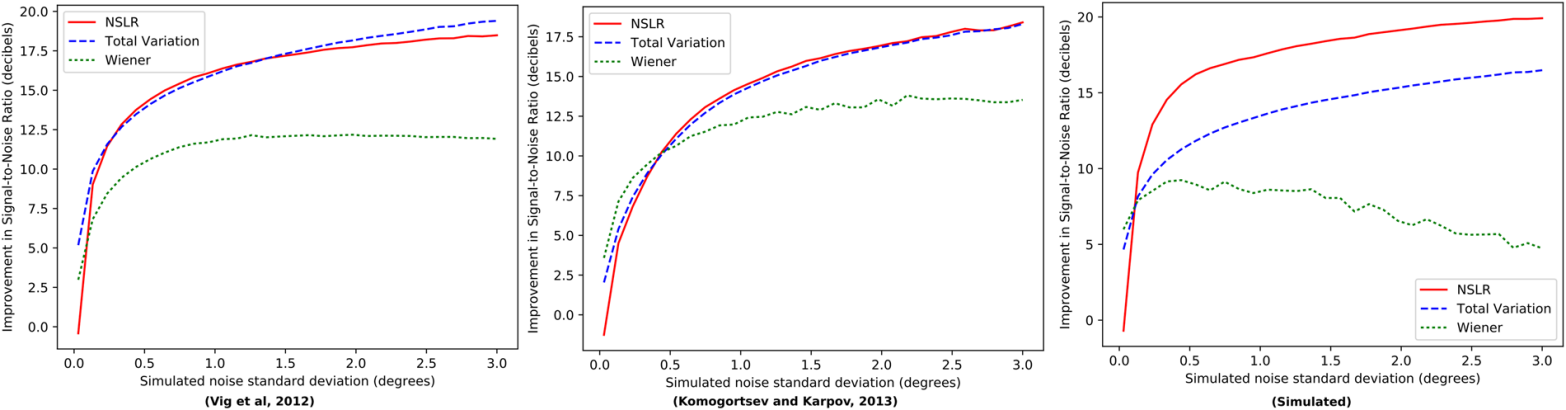
Denoising performance (higher is better) of NSLR in comparison to the Total Variation and Wiener filters at different noise levels. Results are shown for human data and one simulated dataset. Total Variation and Wiener parameters were numerically optimized against each recording (see Denoising benchmarking). NSLR was run using its default parameters (see Measurement noise and segmentation penalty parameters).

**Figure 3:**
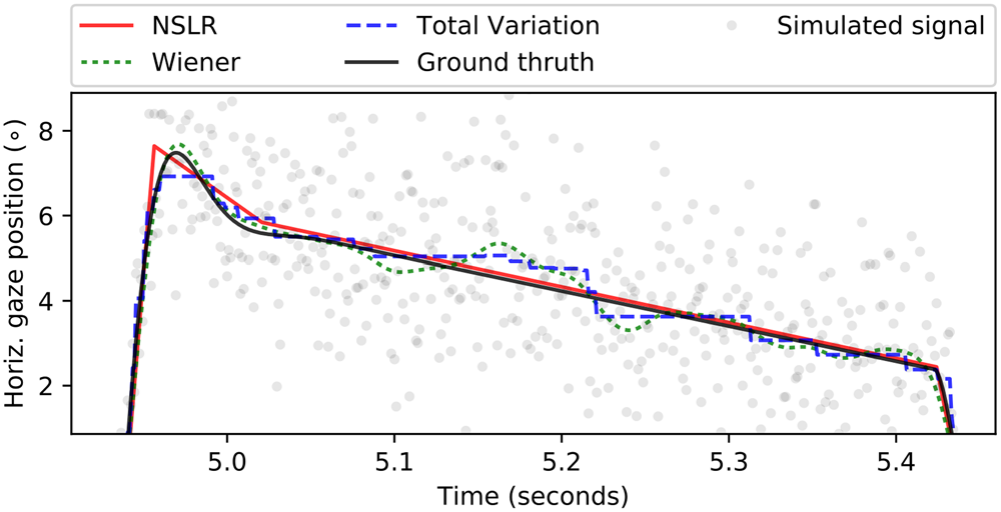
Sample time series of a simulated linear smooth pursuit (solid black line) separated by saccades, and the denoising results of the three benchmarked algorithms with simulated sampling noise level of 1.5° (gray data points). NSLR (solid red line) identifies four segments. Note that it approximates the intersaccadic slow movement interval as two separate linear segments, with the first corresponding to the post saccadic oscillation. Total Variation (dotted blue line) and Wiener (dotted green line) filters reduce the measurement error considerably as well. Total Variation produces an approximately piecewise constant signal, which is characteristic to this algorithm. Wiener filter recovers the PSO well but exhibits ringing effects during the more linear part. Supplementary Figures S4–S6 show NSLR with different simulated noise levels using the simulated data.

Denoising performance measured using Improvement in Signal-to-Noise Ratio (ISNR), which is plotted for each algorithm in Fig. 2. At very small noise levels (σ ≲ 0.1°), NSLR has the weakest performance on all datasets. This is likely due to linear approximation error of the gaze signals’ nonlinearity dominating over denoising on very low noise levels. In the non-simulated datasets, some additional nonlinearity is introduced by the original recording’s measurement noise (see Human eye movement data). On higher noise levels, NSLR and the per-recording optimized Total Variation filter have a similar denoising performance, with NSLR slightly stronger on moderate noise levels (0.3° ≲ *σ* ≲ 1.5°). Apart from very low noise levels, the Wiener filter has clearly the worst performance. This is likely due to linear time-invariant filters’ inherent difficulties with abrupt changes in a signal’s spectral features during fast saccade movements. Figure 3 shows an example of the time series behavior of the different algorithms.

## Event Identification

Event identification in the eye tracking literature refers to methods that classify a sequence of raw data samples from an eye tracker – usually a time series of eye horizontal and vertical rotations – into discrete event types (event detection), and determine the begin and end points of oculomotor events, ie successive samples belonging to the same type (segmentation)^10^. In static scenes, the event classes are usually fixations and saccades. In dynamic scenes, or ones including observer movement, fixations are either replaced by or augmented with the smooth pursuit eye movement event class. This immediately causes problems for standard methods developed mainly for static stimuli^11^. Also, methods that ignore post-saccadic oscillations (PSO’s) face problems. Whether the PSO is placed to the end of a saccade or the beginning of the fixation/pursuit clearly affects effect durations^25^. Also if gaze velocity is used as a feature for identifying saccades (and differentiating them from smooth pursuit), then it makes a difference whether the relatively high-speed observations during the transition from the saccade to the slow phase movement are placed into the fixation/pursuit event, or treated as a separate event type.

Traditionally, event detection was done by hand. Classification relying on the intuition of an experienced researcher is still taken to represent a “gold standard” or ground truth in many cases^11,26,27^. A number of algorithmic methods have been developed, the advantage of which is that they can make use of more explicit and reproducible rules and criteria, and, hence, offer in principle potential for greater transparency and reproducibility of results. In practice, however, manufacturer provided algorithms are typically not documented or available as open source code, and the generality and agreement between algorithms does not meet the level of human experts’ labeling^28,29^. To ensure the replicability of results across groups using different eye-tracking equipment and physical apparati, the development of more standardized and, most of all, publicly available methods for analyzing the eye tracker signals are desirable.

Another drawback of many methods in wide use today is that a number of parameters have to be adjusted by the user. This includes not just parameters for filtering the raw signal but also for specifying the basic properties of each event type (such as minimum acceleration of a saccade, maximal dispersion during or minimum duration of a fixation). These event detection parameters need to be adjusted on the basis of empirical assumptions about the dynamics of the event of interest, while simultaneously considering sampling frequency and measurement noise, and effects of the filtering step. Unfortunately, no hard and fast rules exist, and parameters are often chosen by trial and error (or simply copying values from the literature) which is counter the to the desiderata of reproducibility and transparency.

### Event identification with a Hidden Markov Model

The segmentation produced by NSLR can be interpreted as a series of discrete oculomotor events, with end boundaries. For traditional event identification that aims to parse the signal into events of different “types”, all that remains is to classify each segment into one type.

We will demonstrate the method using the annotated dataset by Andersson *et al*.^11^, allowing direct comparison of our method to human labeling. We will identify all four classes annotated by the human experts (fixation, saccade, post-saccadic oscillation and smooth pursuit). For this purpose, we use a four-class Hidden Markov Model classifier with observations modeled using multivariate Gaussian distributions of log-transformed velocities and Fisher-transformed cosine of the angle between subsequent segments.

The XY angle (in screen coordinates) between subsequent samples has been used in differentiating between smooth pursuits and fixations^12^, but is also useful for identifying post saccadic oscillations. In a PSO the gaze position rapidly oscillates at the end of a saccade with roughly 180-degree reversals. This property can be used to separate PSO’s from the preceding saccade and ensuing fixation/pursuit. Including PSO’s in the model also improves performance on identifying saccades, fixations, and pursuits themselves.

Traditional event detection methods rely on sample-to-sample velocities for classification and a separate pre-filtering step. Using the segmented signal has two major advantages over such methods. The velocity and amplitude estimates are more robust and based on a method that uses raw, unfiltered eye position signal, thus preserving velocity information in the data. Also, features of whole segments (e.g. duration or angle) rather than just individual samples can be used as a classification feature.

**Figure 4:**
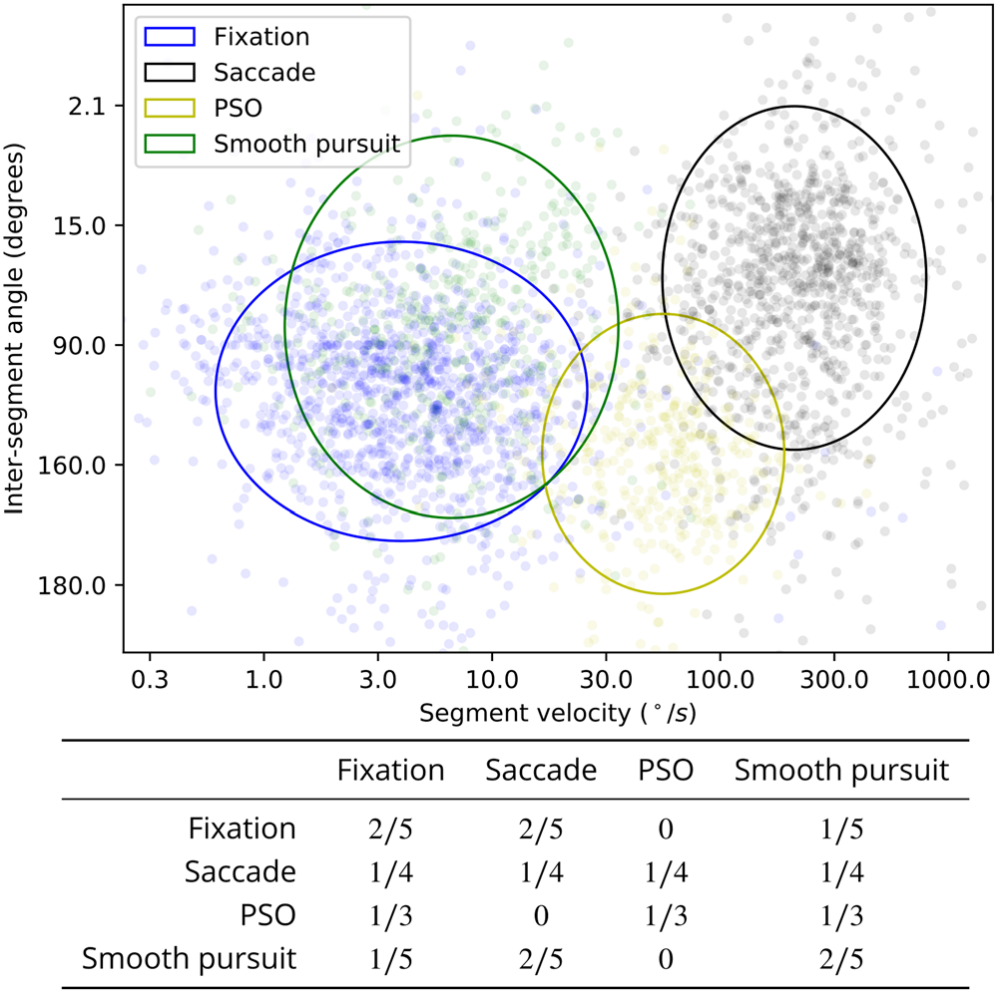
Parameterization of the event classifier. **Top:** Segment classification (human) based on the annotated dataset of^11^, and 90% highest density regions of the per-class estimated distributions. Each datapoint corresponds to one segment given by NSLR. **Bottom:** Transition probabilities of the four-state HMM.

Figure 4 shows the log gaze speed and inter-segment angle values for each segment identified by NSLR. The color of each datapoint indicates the event class assigned (to the majority of samples in the segment) by a human annotator. To estimate the per-class feature distributions, we first classify label each segment using the human classification the dataset by Andersson *et al*.^11^. Using such classified samples, each class’ distribution is estimated using sample mean and variance.

As can be seen from the figure, the distributions of the segments divide the feature space into three clusters, corresponding to saccade, PSO and fixation/pursuit, and there is still substantial overlap between the clusters. To remedy some of the overlap, an HMM with transition probabilities given in Fig. 4 were used. Transition probabilities are mostly used to enforce that post-saccadic oscillations can only occur after saccades, and a saccade cannot directly follow a PSO. Also, direct transitions between smooth pursuit and fixation classes are given lower probabilities than the “merging” of successive fixations or pursuits. This formalizes a kind of “labeling inertia”: a non-saccadic segment between fixations is more likely to be classified as a fixation, and a non-saccadic segment between smooth pursuits is more likely to be a smooth pursuit. To avoid the Markov model switching between classes in contiguous non-saccadic segments the transition probabilities between classes are set at half the within-class transition probability (1/5 vs. 2/5). All other feasible transitions are given equal probability to transition into any feasible state. The initial state probability is uniform 1/4 across classes.

The transition probabilities could also be directly estimated from the data. However, we opted for the simple state transition model described because observed transitions depend greatly on the dataset used: e.g. if the majority of the recordings are of static stimuli, transitions into smooth pursuits from any class are rare just because they are not observed. The choice to introduce the “labeling inertia” was partly inspired by examining the human labeling, but also due to the fact that very low-velocity segments can be detected during (slower) smooth pursuits due to measurement noise.

Per-segment classes are estimated by finding the maximum likelihood event sequence under the model using the Viterbi algorithm. All segments in the data set are thus classified to maximize the overall likelihood (rather than going through the data series one segment at a time with the previous segment fixed in the previous step). Sample-level classification is attained by assigning all samples belonging to a segment into the segment’s class.

### Classification compared to human labeling results

The agreement with manual human event detection was assessed using the human annotated dataset by Andersson *et al*.^11^, which also includes classifications of 10 different algorithms with parameters tuned for this dataset. For NSLR, the feature distributions of the classes were estimated from the human labeled data as described above.

Table 1 lists Cohen’s kappa values for the human raters, NSLR, and the ten algorithms. For four-class classification, the average per-class Cohen Kappa values between the two human raters and NSLR indicate high agreement (0.82) for saccades, and moderate agreement for fixations (0.51), smooth pursuits (0.43) and post-saccadic oscillations (0.53), with overall Kappa of 0.43. Out of the 11 algorithms, this is the strongest agreement for all classes except for post-saccadic oscillations, on which NSLR-HMM is second to the LNS algorithm specifically designed to detect post-saccadic oscillations^30^.

**Table 1:** Cohen’s Kappa values for algorithm versus human agreement for NSLR and the ten algorithms included in the study of^11^. A dash indicates that the algorithm doesn’t (explicitly) use that class. Best performing algorithm for each class is in boldface.

Class	Human	NSLR-HMM	LNS	IVT	EM	IHMM	NH	IKF	IMST	IDT	CDT	BIT
Binary	0.90	**0.82**	0.81	0.76	0.74	0.70	0.67	0.58	0.55	0.47	0.42	0.41
Saccade	0.90	**0.82**	0.81	0.76	0.74	0.70	0.67	0.58	0.55	0.47	—	—
Fixation	0.81	**0.51**	—	0.31	—	0.32	0.20	0.32	0.10	0.09	0.26	0.32
Smooth pursuit	0.79	**0.42**	—	—	—	—	—	—	—	—	—	—
PSO	0.73	0.53	**0.64**	—	—	—	0.24	—	—	—	—	—

Of the included algorithms, NSLR-HMM is the only one that includes all four classes, and only two others include post-saccadic oscillations. To include all of the algorithms for an overall benchmark, we study saccade versus slow eye movement binary classification by collapsing all slow eye movements (fixations, smooth pursuits and post-saccadic oscillations) into a single class. In binary classification, NSLR has the highest agreement with the human raters with Kappa of 0.82 although LNS has similar Kappa score of 0.81.

**Figure 5:**
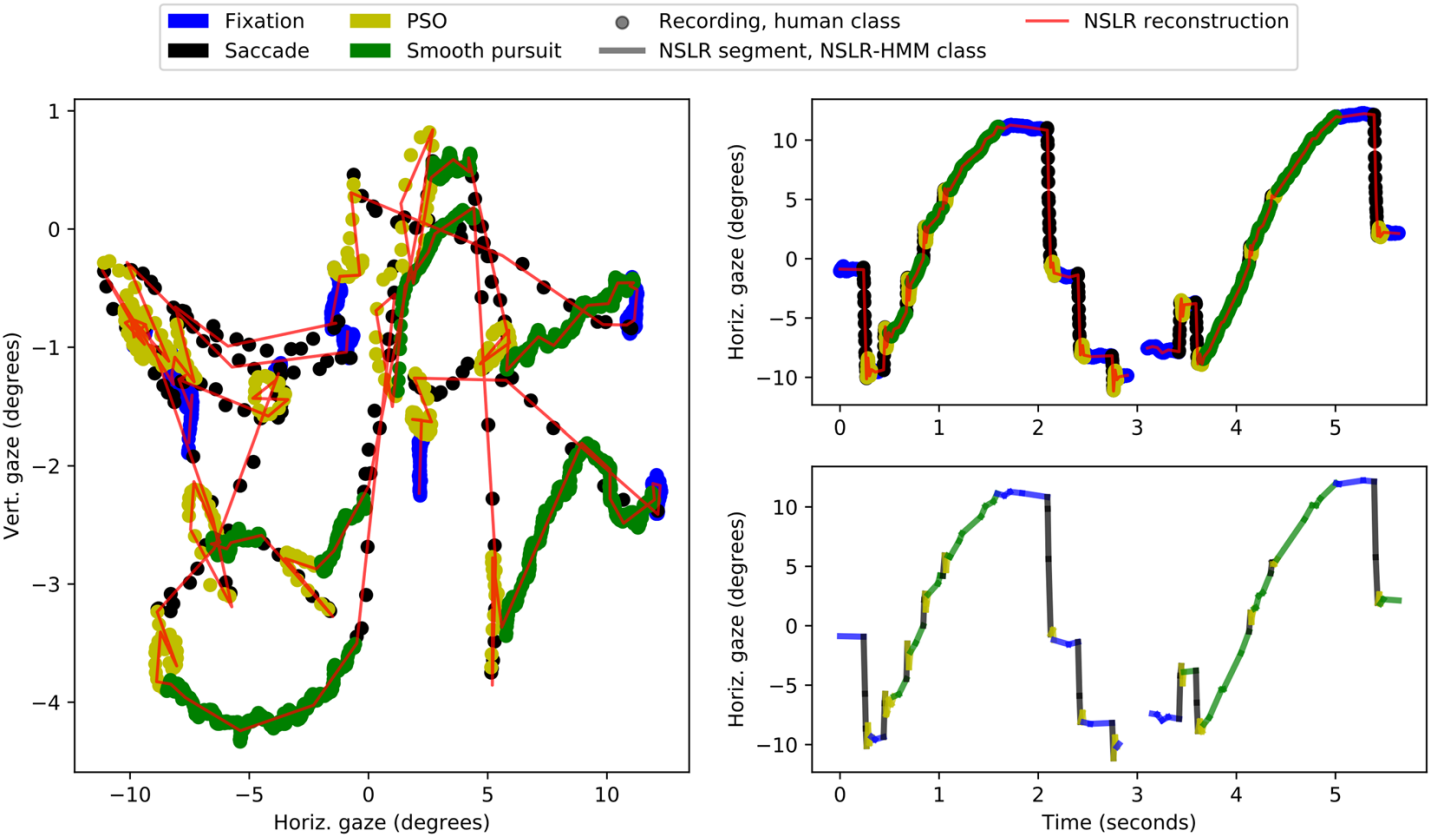
Sample-level data from a gaze recording where the participant is watching a video of a triple jumper passing in front of the camera. For a more detailed view, please see the Supplementary Figure S7. Data and human labeling from^11^. Left: Vertical and horizontal recorded gaze position samples. Dot colors indicate human coder four-way classification, the thin red line shows the piecewise-linear NSLR segments. Top right: Time series of the vertical gaze coordinates and NSLR reconstruction overlaid. Color coding as in the left panel. Bottom right: Individual segments extracted by NSLR, color coded to show the four-way classification of the segments by NSLR-HMM.

The majority of total classification disagreement in the four-class classification can be attributed to the difficulty in distinguishing between fixations and smooth pursuits. This can be expected by examining the feature distributions of the classifier (see Fig. 4), where the two classes overlap considerably. One such disagreement with a human classification can be seen in Fig. 5, where a rather low-velocity segment is classified as a fixation by the human coder but as a smooth pursuit by NSLR-HMM. Examples of NSLR-HMM classifications using real-world noisy data can can be seen in Supplementary Figures S8–S11.

## Future Directions

One obvious idealization the method uses is the assumption of linear (in the XY plane) form of eye movements, which leads to some reconstruction inaccuracy during saccades, and forces the method to reconstruct curved pursuit movements from successive linear segments. Nonlinear segments could probably improve accuracy, but it’s unclear if such can be even approximated in linear time (the segmentation problem becomes the full penalized spline problem). And, as shown, despite the clearly counterfactual idealization the linear segmentation actually does a very good job.

Of course, we have here demonstrated the method only on human data collected during watching naturalistic pictures and movies. These datasets have the benefit of being originally measured with very high accuracy, giving us an empirical ground truth to compare the performance under added simulated noise against. One obvious next step is to formally show how the method works for fully naturalistic free-head-movement locomotor real-world data. For a qualitative view on how NSLR-HMM performs on such data, please see Supplementary Figures S8–S11. As can be seen from these samples, naturalistic data tends to have outlier measurements, which violate our assumption of gaussian noise. Making the segmentation robust to such outliers should be rather straightforward by including an “outlier hypotheses” in the segment estimation (such was already used in a previous version of the method for very noisy driving data^31^).

To benchmark classification with more naturalistic data, large annotated datasets with multiple event types labeled by experts would be needed. Here, though, there is an additional conceptual complication: the way oculomotor events are defined and identified can be conceptually quite different in naturalistic and laboratory tasks. Perhaps the main mismatch is that “fixations” are usually discussed in terms of traditional laboratory settings, i.e. static environments with usually fixed observer position. Such settings lead to a signal that is approximately constant during fixations which are separated by fast saccades. In naturalistic settings such signal almost never occurs; even with static environment, the observer usually moves, which leads to gaze signal dominated by “slow eye movements”, or smooth pursuit and compensatory eye movement events. From the *task analysis perspective*, these are fixations to scene targets, but from the traditional *oculomotor perspective* such eye movements are usually seen as a “separate” smooth pursuit case^5^. How and whether to separate fixation from smooth pursuit remains an open conceptual issue that impacts the desiderata of event identification. As the function of a classification is to reflect this conceptual separation, not vice versa, the algorithmic classification results should mirror, or at least be interpreted from, the task analysis perspective.

The event identification accuracy could be improved by including apriori assumptions about durations of the different event types. One way could be using the segment durations as a classification feature, but we decided against this as the segment durations vary also due to recording noise, sampling rate and microevents, thus hindering generalizability. A more principled and generalizable way would be to use a Hidden Semi-Markov model, which support more flexible duration distributions, but at the cost of significantly higher computational complexity^32^.

The current formulation of NSLR-HMM requires human labeled data for the classifier parameter estimation. As it’s formulated as a Hidden Markov Model, the parameters could be automatically estimated using standard HMM reestimation methods like the Baum-Welch and the Viterbi reestimation algorithm, as is often done with the I-HMM identification algorithm^10^. However, in our informal experiments such reestimation tended to converge to rather bad solutions, which we hypothesize at least partly to be due to the weak separation of fixations and smooth pursuits in the feature space (see Fig. 4). Should this be the reason, the reestimation could be made more robust using a hierarchical model, where the fixation/pursuit would be estimated first as a single class, and the separation between these classes would be estimated independently of the other classes (if a strict fixation/pursuit separation is desired).

The most principled and likely most effective development to increase an accuracy of both denoising and event identification would be to identify the segments and events in an integrated manner. This would involve integrating the prior knowledge of the durations and segment slopes of the different classes into the segmentation algorithm. Such formulation would, however, violate the PELT method’s assumptions even further and could prove difficult to do in a performant and accurate manner.

The presented formulations of denoising and event identification are based on finding an approximate global optimum based on the whole data, and thus are not directly applicable to online use. NSLR could be used for zero-latency denoising quite simply by greedily estimating the current point based on the currently viable hypotheses, for example by taking the predicted value of the current most likely hypothesis, or a likelihood-weighted average of all hypotheses. Somewhat better performance would be expected if some latency is tolerated and NSLR would be formulated e.g. as a fixed-lag smoother. However, behavior and performance of such usage should be studied empirically, as effects of the hypothesis pruning and greedy continuity enforcement are not understood. Furthermore, the resulting estimate would lose the piecewise linear structure, which introduces difficulties for the segment-based HMM classification.

Finally, it should be repeated that NSLR finds only an approximation of the optimal segmentation. The ramifications of our greedy approximation are not currently understood, although it almost certainly causes some bias in the estimate. The future inquiry should at least study these biases, and ideally, find methods to reduce or even eliminate them by an optimal algorithm.

## Discussion

We have introduced here a method for eye-movement signal analysis, NSLR-HMM, that has some novel and desirable features compared to the state of the art. The most important novelty lies in the way the workflow of the algorithm differs from the more the traditional workflow for event identification, where the signal is usually pre-filtered before event identification, and event detection and segment boundaries are based on a sample-wise application of heuristic rules (such as hard event duration or eye speed thresholds).

With NSLR-HMM event identification begins with segmentation that simultaneously denoises the signal and determines segment boundaries. What is more, NSLR is not performed by proceeding one sample at a time but based on a search for an approximately optimal segmentation of an entire time-series measurement. Our implementation can process tens of thousands of samples per second on a normal laptop PC, regardless of the time series length.

An important advantage is that the signal need not (and should not) be pre-filtered. Thus, the user is not required to make critical preprocessing choices that potentially affect the appropriate parameter choices in subsequent event identification. (For example, low-pass filtering leads to a lowering of observed gaze speeds, which affects the results of velocity-based thresholding algorithms). Of course, such choices of event identification threshold values themselves would also make substantial empirical assumptions (e.g. minimum fixation duration), which become incorporated into the reduced event time series data. When these choices are based on accepted practice (values copied from previous studies) there is a danger that such assumptions will not be tested against the accumulating data, and will propagate in the literature without being proper replications. Clearly an undesirable state of affairs — which can be much mitigated by adopting our analysis method that is not based on prefiltering and thresholding, and does not make the user face the complex and poorly understood problem of matching filtering and event identification parameters.

The classification step does require empirical assumptions about the underlying dynamical characteristics of the eye movements one is interested in, and we use human labeling data to estimate the parameters for the classifier. However, these assumptions are not expressed in the form of a few local rules (e.g. sample to sample gaze velocity). Human expert classifiers do not follow such rules, but observe an overall pattern in the signal and rely on their experience which may not be reducible to a small number of rigid rules. This makes us suspect that any algorithm that relies on such local rules rather using more global information in the signal is unlikely to be able to match a human labelling very well, at least when the algorithm cannot rely on highly restrictive assumptions about subjects’ gaze behavior, but needs to be able to classify a large number of eye movement types (four in our case).

## Conclusions

For over a hundred years, the oculomotor system has served as a model system for studying motor control, and as a window into perception, cognition and other higher brain functions. Over the past 25 years, a wealth of studies on gaze behavior using more naturalistic stimuli and even real-world tasks has delivered important insight into oculomotor control in more complex settings than traditional oculomotor experiments^33,34^. Especially in difficult conditions, such as mobile eye tracking in real world tasks, event detection methods generally work on a prefiltered signal to avoid spurious detected events due to measurement noise but this leads to its own problems.

In laboratory conditions, the measurement noise is usually low and a subtle low-pass filtering usually suffices for most cases. However, for more challenging measurement environments and when studying more minute aspects of eye movements, such as microsaccades and subtler smooth pursuits, finding a filter that eliminates the noise but retains important signal characteristics can be demanding^15^.

Our approach that unifies signal denoising and segmentation, and treats segment classification (and its correspondence to human labelling) as a separate problem, has the potential to remove many sources of error and aspects of “black art” from eye tracking signal analysis, encourage a more conceptually elegant and clear view of the event identification problem itself, and, hopefully help in the methodological and theoretical unification of the traditional laboratory oculomotor research and the study of complex gaze behavior “in the wild”.

## Methods

performance in denoising and classification, we use three published human eye-movement datasets available^11,24,35^. In addition, we generate simulated eye movement data for estimating the parameterization of our method and for assessing the filtering performance in an ideal case where the absolute ground truth (gaze position at each moment in time) is known and follows the model. The human datasets include both static and dynamic stimuli and are recorded with high-quality equipment in laboratory conditions.

Two of human datasets^24,35^ are used for evaluating NSLR’s denoising performance. Denoising performance is assessed using simulated Gaussian noise and measured using Improvement in Signal-to-Noise Ratio (ISNR), which is a rather straightforward transformation from the mean square errors of the denoising result and simulated noise. The reference algorithms are numerically optimized against the “ground truth”, ie the recording without additional noise.

The third dataset^11^, which we use for evaluating the eye-movement classification performance, includes human four-way labeling by two coders. Ten different algorithms tuned for this dataset are compared with NSLR-HMM. Eye-movement classification is assessed using per-sample agreements with the human coders and measured using Cohen’s Kappa.

### Human eye movement data

#### Andersson *et al*

**Figure 6:**
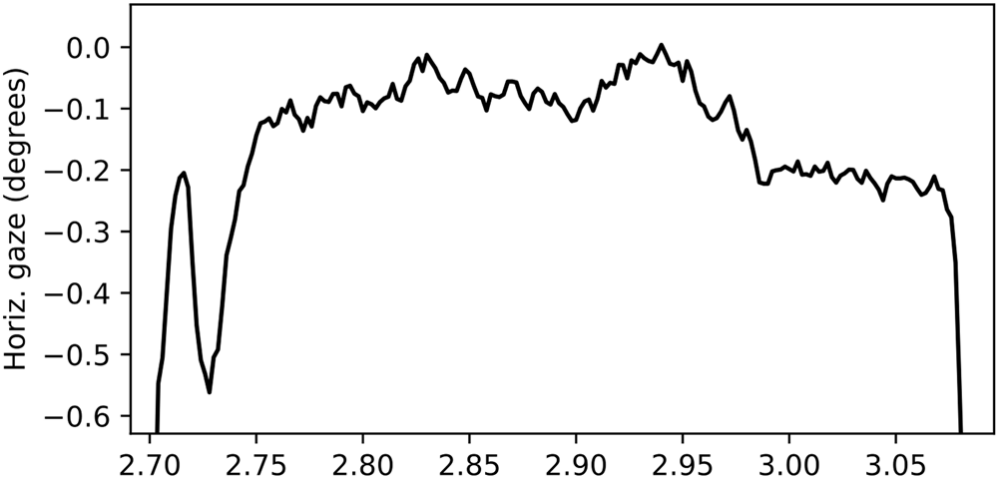
A sample recording of a single fixation from the dataset by Andersson *et al*.^11^.

The dataset by Andersson *et al*.^11^ was recorded with SMI HiSpeed 1250 at a 500 Hz sampling rate, with reported noise RMSD of about 0.03°, which translates to about 0.02° standard deviation per axis. The recording was binocular, but only the signal from the right eye was used. The signal was prefiltered using the equipment vendor’s implementation of the Bilateral Filter. Unfortunately, details of the implementation don’t seem to be documented by the vendor. See Fig. 6 for a sample of the signal.

The stimuli used include static images, video clips and moving dot targets. We include all of the recordings that were used in the evaluation of^11^, which totals in 35 recordings from 17 different participants.

Each recorded gaze sample is classified by two human coders into one of six classes: saccade, fixation, smooth pursuit, post saccadic oscillation, blink or undefined. The samples assigned to the latter two classes of either rater were included in the algorithms’ classifications but omitted when calculating the Cohen’s Kappa values. In addition, the dataset includes per-sample classification using ten different algorithms tuned in various degrees to the dataset. The algorithms and their parameterizations are documented in^11^. The dataset is available online at https://github.com/richardandersson/EyeMovementDetectorEvaluation.

#### Vig *et al*

**Figure 7:**
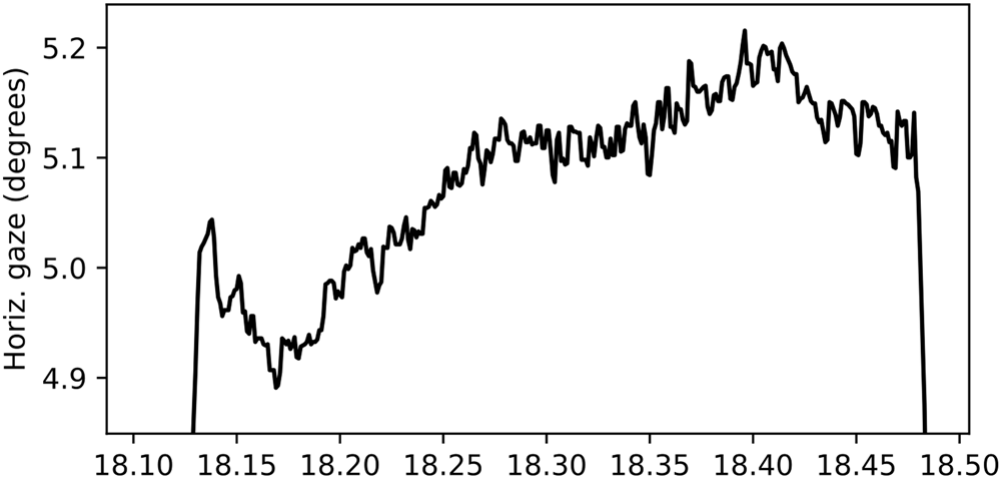
A sample recording of a single fixation from the dataset by Vig *et al*.^24^.

The dataset by Vig *et al*.^24^ consists of recordings of five participants watching movie clips from a video dataset^36^. The eye movements were recorded using SR Research EyeLink 1000 with sampling rate 1000 Hz. The recording was binocular and the resulting signal is the estimated gaze position using both eyes. The recording noise level is not reported, but we estimate its standard deviation to be around 0.03° per axis (see Fig. 7). No prefiltering step was reported.

The total data consists of 1706 recordings per subject, but some of these were omitted due to a high level (over 20%) of outliers. For the denoising benchmark, we randomly selected 100 recordings out of recordings lasting over 20 seconds. The dataset is available online at https://doi.org/10.6084/m9.figshare.93951.v4.

#### Holland and Komogortsev

**Figure 8:**
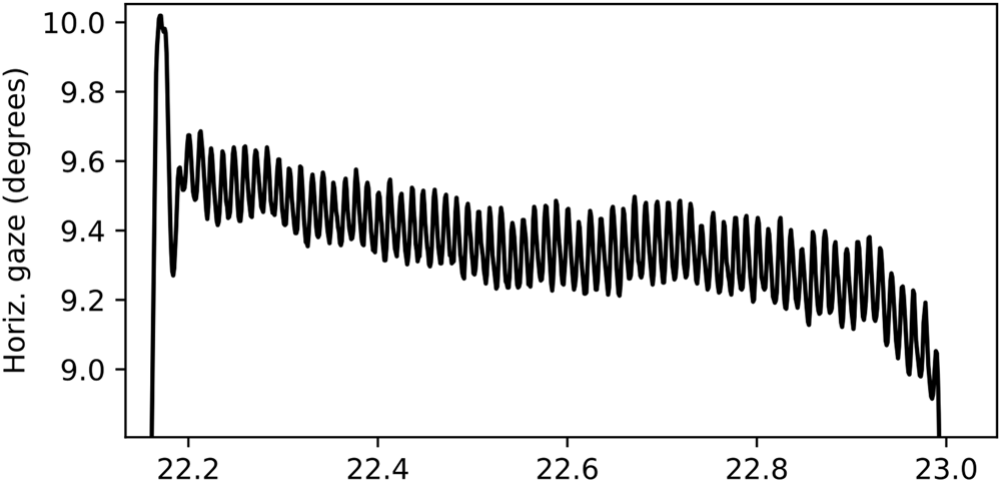
A sample recording of a single fixation from the dataset by Holland and Komogortsev^35^.

We used the “Dataset II” from the dataset by Holland and Komogortsev^35^. It was recorded using the EyeLink 1000 at 1000 Hz sampling rate using the monocular mode. The used stimuli were dot targets, which moved stepwise to induce saccades and linearly to induce smooth pursuits. The signal was prefiltered using the vendor’s implementation of the algorithm described by Stampe^12,37^. The signal seems to have high-frequency oscillatory-type correlated noise, which may be ringing introduced by the prefiltering. The noise level is not reported, but we estimate its standard deviation to be around 0.1° (see Fig. 8). The dataset is available online upon request at http://cs.txstate.edu/ ok11/embd_v2.html.

### Simulated eye movement data

For parameter estimation, we generated random eye-movement sequences using a simplified eye-movement model. We simulate scenarios where a participant follows a target with their gaze. A scenario is parameterized by maximum fast phase amplitude *a* ^*^, maximum slow phase duration *d* ^*^ and maximum slow phase velocity *v* ^*^. To generate the gaze target signal, after each slow phase, the signal jumps from the current position to random direction with amplitude drawn from uniform distribution *u*(0, *a* ^*^). After the jump, it starts a slow phase with duration drawn from *u*(0, *d* ^*^) to a random direction with velocity drawn from *u*(0, *v* ^*^).

**Figure 9:**
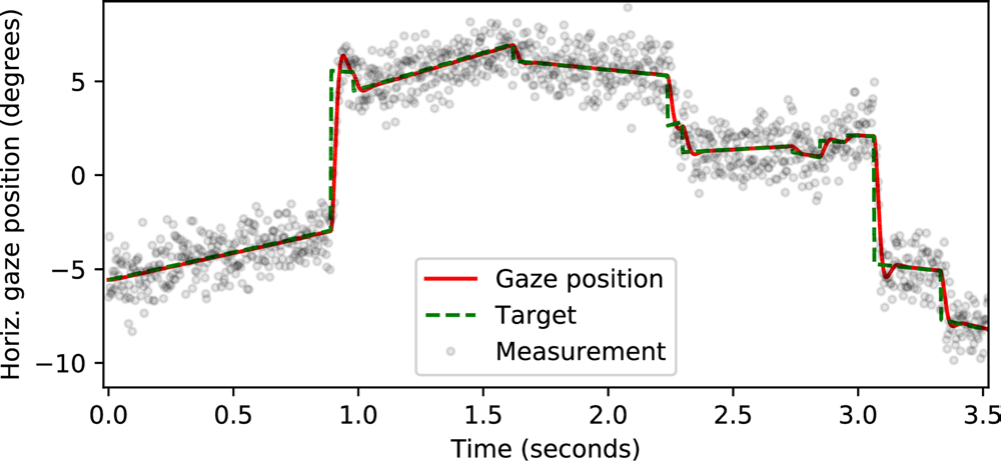
A sample time series of a simulated recording, where gaze (solid red line) tracks a target (dotted green line) that jumps from place to place producing rapid saccadic eye movements, and in between moves with a slow (down to zero) velocity producing slow movements/fixations. Noise is added and the signal resampled to produce simulated measurement data. Maximum fast phase amplitude a* = 10°, maximum slow phase duration d* = 1*s*, maximum slow phase speed v* = 5°/*s* and noise level *σ* = 1.0°.

To approximate eye-movement characteristics, we use a simple damped oscillator model^38^ with independent vertical and horizontal axes, and use position difference from the target signal as driving force: $\ddot{\overrightarrow{x}}=K(\overrightarrow{x}^{\prime} -\overrightarrow{x})-D\dot{\overrightarrow{x}}$, where $\ddot{\overrightarrow{x}}$ is the (output) eye acceleration, $\overrightarrow{x}^{\prime}$ is the target position, $\overrightarrow{x}$ is the eye position, $\dot{\overrightarrow{x}}$ is the eye velocity and *K*Â and *D*Â are scalar constants. See Fig. 9 for an example of the simulated data.

**Figure 10:**
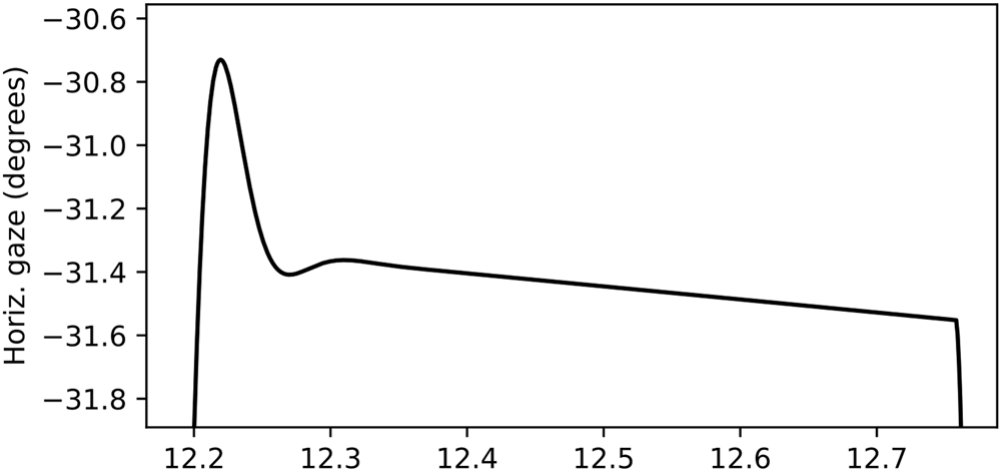
A sample of a single simulated fixation (or very slow smooth pursuit).

The parameterization of the eye model (*K* = 6000, *D* = 90), was selected so that the produced eye movements approximately follow the peak velocity, saccade duration and peak acceleration of the “saccadic main sequence”^39^. Completely faithful replication of the empirical main sequence results isn’t possible, as the model form forces the peak velocity and acceleration to be linear wrt. saccade magnitude, and the saccade duration is constant regardless of the magnitude. The produced signal can nevertheless be taken as quite a reasonable approximation, for our specific purpose of calibrating parameters for the NSLR algorithm. See Fig. 10 for a detailed view of a simulated fixation/slow smooth pursuit.

The simulated signal was generated at 1009 Hz and downsampled using linear interpolation to simulate different eye tracker sampling rates. Each scenario was 2 minutes in duration. Axis-independent Gaussian noise was added to the generated signal to simulate different levels of measurement noise.

### Benchmarking methodology

#### Denoising benchmarking

The denoising performance was measured using Increase in Signal-to-Noise Ratio:

1 $${{\rm{ISNR}}}_{{\rm{DB}}}=10{\mathrm{log}}_{10}\frac{\sum _{i}|{\overrightarrow{x}}_{i}-{\overrightarrow{g}}_{i}{|}^{2}}{\sum _{i}|{\overrightarrow{x}}_{i}-{\overrightarrow{\hat{g}}}_{i}{|}^{2}},$$

where ${\overrightarrow{x}}_{i}$ is the measured (noise contaminated) gaze position, ${\overrightarrow{g}}_{i}$ is the true gaze position and ${\overrightarrow{\hat{g}}}_{i}$ is the reconstructed gaze position at measurement index *i*. Before introducing the data to the algorithms, all samples marked as outliers in the dataset were removed and replaced by linearly interpolating the signal using the nearest two non-outlier samples. The error in the interpolated samples was not included in the calculation of the ISNR values. Furthermore, 256 first land last values were omitted from the error calculation to avoid extraneous error due to boundary effects for the Wiener filter.

The Wiener filter convolution kernel was derived by taking the inverse Fourier transform of the ratio between estimated cross power spectral densities (CSD) of the noise contaminated and uncontaminated signals for each recording. The CSDs were estimated using the Welch method, as implemented in scipy.signal.csd^40^, using a 256 sample Hamming window.

For the Total Variation filter, we used the implementation by^20^, available online at https://github.com/albarji/proxTV. The regularization parameter was optimized against the uncontaminated data of each recording using the L-BFGS-B algorithm as implemented by scipy.optimize.minimize^40^.

NSLR used the default parameterization (see Measurement noise and segmentation penalty parameters) without access to the uncontaminated data.

#### Gaze event identification benchmarking

Gaze event identification was assessed by measuring agreement with human coder identification by^11^. The agreement was operationalized as Cohen’s Kappa values, as implemented by sklearn.metrics.cohen_kappa_score^41^. Prior to calculating the agreement scores, all samples that either of the human coders labeled as outliers (“blink” or “other”) were omitted. The reported Kappa value for the algorithms is the arithmetic mean of the algorithm’s agreement to both human coders.

## Electronic supplementary material

Supplementary Information
