# Supplementary material for: A new and general approach to signal denoising and eye movement classification based on segmented linear regression
Source: Sci Rep. 2017 Dec 18;7:17726. doi: 10.1038/s41598-017-17983-x (PMC5735175; doi:10.1038/s41598-017-17983-x)
Supplement: Supplementary file 1 — Supplementary Information [file 41598_2017_17983_MOESM1_ESM.pdf]

Supplementary information:
A new and general approach to signal denoising and eye movement classification based on segmented linear regression

Jami Pekkanen[1,*] and Otto Lappi[1]

[1]University of Helsinki, Cognitive Science, Helsinki, 00014, Finland
[*]jami.pekkanen@helsinki.fi

Note: Naive Segmented Linear Regression

Formal derivation

Given a time series of gaze position measurements $\mathbf{g}[i] = (x[i], y[i])$ of true gaze positions $\mathbf{G}(t) = (X(t), Y(t))$ from time t_0 to t_e sampled at time instants $t[i], i \in I$, the gaze position function is approximated by m linear functions:

$$
\begin{aligned}
\hat{\mathbf{g}}_J(t) &= \mathbf{a}_0 t_0(t) + \mathbf{b}_0, && t[j_0] \leq t < t[j_1 - 1]\\
\hat{\mathbf{g}}_J(t) &= \mathbf{a}_1 t_1(t) + \mathbf{b}_1, && t[j_1] \leq t < t[j_2 - 1]\\
&\vdots && \vdots\\
\hat{\mathbf{g}}_J(t) &= \mathbf{a}_m t_m(t) + \mathbf{b}_m, && t[j_m] \leq t < t_e,
\end{aligned}
$$

where $j \in \{0, 1, \ldots, m\} \equiv J$ is the index for the segment start points, $\mathbf{a}_j = (a_{xj}, a_{yj})$ $\mathbf{b}_j = (b_{xj}, b_{yj})$ are the slopes and intercepts for the j:th segment's x and y coordinates and $t_j(t) = t - t[j-1]$. The first segment start j_0 corresponds always to the first measurement in the series at $t[0]$ and $t[-1] = t[0]$.

The measurement errors are assumed to be axis-independent and Gaussian distributed:

$$
\begin{aligned}
\varepsilon[i] &\equiv \mathbf{G}(t_i) - \mathbf{g}[i] \sim \mathcal{N}(\mathbf{0}, \Sigma)\\
\Sigma &= \operatorname{diag}(\sigma_x^2, \sigma_y^2).
\end{aligned}
$$

Segment durations are assumed to be exponentially distributed, with mean duration τ. Thus, the probability of a new segment starting between two measurements $i-1$ and i is the probability that the exponential distribution "waiting time" is less than the time between the measurements. To simplify the final objective function, we approximate the logarithm of the split probability function as its first order truncated Maclaurin series expansion, which is simply $log(\tau^{-1}\Delta t[i]) + O(\tau^{-1}\Delta t[i])$), and provides a reasonable approximation for eye movement signals, where usually $\tau^{-1}\Delta t[i] \ll 1$:

$$
\begin{aligned}
\Delta t[i] &= t[i] - t[i-1]\\
\log(P(S \leq \Delta t[i])) &= \log(1 - \exp(-\tau^{-1}\Delta t[i]))\\
&\approx \log(\tau^{-1}\Delta t[i]).
\end{aligned}
$$

Combining these assumptions, we derive an objective function for a segment j:

$$
\begin{aligned}
\mathscr{L}_j &\equiv P(S \leq \Delta t[i_j]) \prod_{i=i_j}^{i_{j+1}-1} f_{\mathcal{N}}(\hat{\mathbf{g}}[i] - \mathbf{g}[i];\, \mathbf{0}, \Sigma)\\
\log \hat{\mathscr{L}}_j &\approx \log(\tau^{-1}\Delta t[i]) + \sum_{i=i_j}^{i_{j+1}-1} \frac{1}{2\pi\sigma_x\sigma_y} - \frac{(x[i] - \hat{x}[i])^2}{2\sigma_x^2} - \frac{(y[i] - \hat{y}[i])^2}{2\sigma_y^2}\\
&= \log(\tau^{-1}\Delta t[i]) + \frac{n_j}{2\pi\sigma_x\sigma_y} - \frac{S_{xj}}{2\sigma_x^2} - \frac{S_{yj}}{2\sigma_y^2}.
\end{aligned}
$$

The objective of NSLR is then to find the segmentation $J^\star$ that maximizes the sum of all per-segment objective functions:

$$\log \hat{\mathscr{L}}(J) \equiv \sum_{j \in J} \log \hat{\mathscr{L}}_j$$

$$J^\star \equiv \underset{J}{\operatorname{argmax}} \log \hat{\mathscr{L}}(J).$$

If we'd assume non-continous segmentation, the well known maximum likelihood estimate for the slopes $\mathbf{a}'_j$ and intercepts $\mathbf{b}'_j$ could be stated as:

$$a'_{xj} = \frac{\sum_{i \in I_j} t_j[i]x[i] - b'_{xj} t_j[i]}{\sum_{i \in I_j} t_j[i]^2} \qquad a_{yj} = \ldots$$

$$b'_{xj} = \frac{(\sum_{i \in I_j} t_j[i])(\sum_{i \in I_j} t_j[i]x[i]) - (\sum_{i \in I_j} t_j[i]^2)(\sum_{i \in I_j} x[i])}{(\sum_{i \in I_j} t_j[i])^2 - n_j \sum_{i \in I_j} t_j[i]^2} \qquad b_{yj} = \ldots$$

However, the non-continuous segmentation is an unrealistic assumption for most data, including eye-movement recordings. The MLE (and its error) for the continuous case, ie $t_j[j]\mathbf{a}^\star{}_j + \mathbf{b}^\star{}_j = t_{j-1}[j]\mathbf{a}^\star{}_{j-1} + \mathbf{b}^\star{}_{j-1}$, can be found in linear time by solving a tridiagonal system, but to our knowledge no method exists to do this *incrementally in O(1) time*, which is a requirement for our O(n) dynamic programming approach. Instead, we enforce the continuity *greedily* so that the next segment is fixed to start from the end point of the previous segment, which is estimated without using the future data:

$$a_{xj} = \frac{\sum_{i \in I_j} t_j[i]x[i] - b_{xj} t_j[i]}{\sum_{i \in I_j} t_j[i]^2} \qquad a_{yj} = \ldots$$

$$b_{xj} = t_{j-1}(t[j])a_{xj-1} + b_{xj-1} \qquad b_{yj} = \ldots$$

$$b_{x0} = \frac{(\sum_{i \in I_0} t_0[i])(\sum_{i \in I_0} t_0[i]x[i]) - (\sum_{i \in I_0} t_0[i]^2)(\sum_{i \in I_0} x[i])}{(\sum_{i \in I_0} t_0[i])^2 - n_0 \sum_{i \in I_0} t_0[i]^2} \qquad b_{y0} = \ldots$$

With the slopes and intercepts known, we can calculate the sum of square errors S_{xj}, S_{yj} and thus the value of the objective function $\log \hat{\mathscr{L}}(J)$:

$$S_{xj} = a_{xj}^2 (\sum_{i \in I_j} t_j[i]^2) + 2a_{xj} b_{xj} (\sum_{i \in I_j} t_j[i]) - 2a_{xj} (\sum_{i \in I_j} t_j[i]x[i]) + b_{xj}^2 n_j - 2b_{xj} (\sum_{i \in I_j} x[i]) + \sum_{i \in I_j} x[i]^2$$

$$S_{yj} = \ldots$$

To find an approximation $\hat{J}^\star$ of the optimal segmentation $J^\star$ we use the dynamic programming approach of[1] with a pruning step inspired by[2]. Please note that due to the greedy enforcement of the continuity, the assumptions of neither method hold, and thus $\hat{J}^\star$ is not necessarily the optimal segmentation even for the greedily continuous case. The dynamic programming algorithm is presented in Implementation of NSLR segmentation.

After the segment endpoint indices $\hat{J}^\star$ are computed, we discard the greedy parameters $\mathbf{a}_j, \mathbf{b}_j$ and instead compute the continuous least squares segmented linear regression using the produced segmentation. The algorithm for this computation is presented in Implementation of NSLR reconstruction.

Implementation of NSLR segmentation

```
# Python implementation of the dynamic programming
# algorithm of NSLR for finding the segmentation.
from numpy import *

# Data structure for a segmentation hypothesis
class Hypothesis:
  def __init__(self, D, j, parent, lik, b):
    # Initialize parent-dependent variables
    self.j = j; self.parent = parent; self.lik = lik; self.b = b;
    # Initialize accumulators to zero
    self.a = None; self.n = 0
    self.t = 0.0; self.St = 0.0; self.Stt = 0.0; self.S = 0
    self.Sx = zeros(D); self.Sxx = zeros(D); self.Stx = zeros(D)

def nslr_segments(ts, xs, noise_stds, mean_duration):
  D = len(noise_stds) # Number of dimensions in the dependent variable
  # Initialize the hypothesis set with a "root" hypothesis
  hypotheses = [ Hypothesis(D, j=0, parent=None, lik=0.0, b=None) ]
  prev_t = ts[0]
  for i, (t, x) in enumerate(zip(ts, xs)): # Iterate over all samples
    dt = t - prev_t; prev_t = t
    for h in hypotheses:
      # Update the accumulator variables
      h.n += 1; h.t += dt; h.St += h.t; h.Stt += h.t**2
      h.Sx += x; h.Sxx += x**2; h.Stx += h.t*x

      if h.parent is None: # Calculate the intercept for the root hypothesis
        if (h.St**2 - h.n*h.Stt) > 0:
          h.b = (h.St*h.Stx - h.Stt*h.Sx)/(h.St**2 - h.n*h.Stt)
        else:
          h.b = h.Sx/h.n

      if h.Stt > 0.0: # Calculate the slope and the residual sums of squares
        h.a = (h.Stx - h.b*h.St)/h.Stt
        S = h.a**2*h.Stt + 2*h.a*h.b*h.St - 2*h.a*h.Stx + h.n*h.b**2 - 2*h.b*h.Sx + h.Sxx
      else:
        S = zeros(D)

      # Update the hypothesis' fitness function
      h.lik += sum(log(1/(sqrt(2*pi)*noise_stds))) + sum((h.S - S)/(2*noise_stds**2))
      h.S = S

    if i == 0: continue # First hypothesis needs at least two samples

    winner = max(hypotheses, key=lambda h: h.lik) # Get the current best hypothesis
    # Create a new hypothesis starting from the winner's endpoint.
    winner_end = winner.t*winner.a + winner.b
    new_lik = winner.lik + log(dt/mean_duration) # Add the split likelihood
    new_h = Hypothesis(D, j=i, parent=winner, lik=new_lik, b=winner_end)

    # Prune hypotheses that have lower fitness than the newly formed hypothesis
    hypotheses = [h for h in hypotheses if h.lik > new_lik or h is winner]
    hypotheses.append(new_h)

  # Find the split points J
  h = max(hypotheses, key=lambda h: h.lik)
  splits = [len(ts)]
  while h is not None: splits.append(h.j); h = h.parent
  return splits[::-1]
```

Implementation of NSLR reconstruction

```
# Produces the tridiagonal matrix coefficients for solving
# the least squares continuous segmented regression
def segment_coefficients(ts, xs, J):
  Smw0 = 0.0; Smw0 = 0.0; Sxw0 = 0.0; Sww0 = 0.0; Smm0 = 0.0
  for k in range(len(J) - 1):
    span = slice(J[k], J[k+1])
    t = ts[span]; x = xs[span]
    dur = (t[-1] - t[0])
    if dur == 0: dur = 1.0
    w = (t - t[0])/dur
    m = 1 - w

    Smw1 = (m*w).sum(); Smm1 = (m*m).sum(); Sxm1 = (x*m).sum()

    p0 = Smw0; p1 = Smm1 + Sww0; p2 = Smw1
    y = Sxm1 + Sxw0
    yield p0, p1, p2, y

    Smw0 = Smw1; Smm0 = Smm1; Sxm0 = Sxm1
    Sww0 = (w*w).sum()
    Sxw0 = (x*w).sum()
  p0 = Smw0
  p1 = 0.0 + Sww0
  p2 = 0.0
  y = 0.0 + Sxw0
  yield p0, p1, p2, y

# Uses the tridiagonal matrix algorithm to solve the
# system generated by segment_coefficients. Returns
# the endpoints of the linear segments at times ts[sis]
def segmented_linear_fit(ts, xs, J):
  bgs = [(0.0, 0.0)]
  for p0, p1, p2, y in segment_coefficients(ts, xs, J):
    b, g = bgs[-1]
    denom = p0*g + p1
    bgs.append(( (y - p0*b)/denom, -p2/denom ))

  endpoint = 0.0
  endpoints = []
  for b, g in reversed(bgs[1:]):
    endpoint = g*endpoint + b
    endpoints.append(endpoint)
  return endpoints[::-1]

# Runs the whole NSLR. Returns times at segment endpoints,
# estimated dependent variable values at endpoints and
# the segment endpoint indices.
def nslr(ts, xs, noise_stds, mean_duration):
  J = nslr_segments(ts, xs, noise_stds, mean_duration)
  endpoints = segmented_linear_fit(ts, xs, J)
  tidx = J[::]; tidx[-1] -= 1
  return ts[tidx], endpoints, J
```

Results: NSLR parameterization

To find the appropriate segment penalty value $\tau_\star^{-1}$ for different recording and stimulus scenarios we generated synthetic eye movement data (see section "Simulated eye movement data" in the main text) using a permutation of parameterizations. For each permutation, the optimal value for τ^{-1} was estimated using grid search. A linear quantile regression (as implemeted by `statsmodels.regression.quantile_regression`[3]) for logit transformed $\tau_\star^{-1}$ was computed for the optimal values using all of the permutation variables as dependent variables:

$$\log \frac{\tau_\star^{-1}}{1-\tau_\star^{-1}} = \beta_{\Delta t} \log \Delta t^{-1} + \beta_\sigma \sigma^{-1} + \beta_a \log a^\star + \beta_d \log d^\star + \beta_v \log v^\star + \beta_1 + \varepsilon,$$

where β are the regression coefficients, ε is the regression error. The variable values used for the permutations and grid search and the resulting coefficients are listed in the table below:

Variable	Symbol	Search range	β
Log penalty	$\log \tau^{-1}$	`linspace(0.0, 20, 10)`	-
Noise level	σ	`geomspace(0.05, 5.0, 5)`	0.5
Sampling rate	Δt^{-1}	`geomspace(30, 500, 5)`	-1
Max. fast phase amplitude	$a_\star$	`linspace(2, 60, 3)`	0.5
Max. slow phase dur.	$d_\star$	`linspace(0.3, 5.0, 3)`	-1
Max. slow phase speed	$v_\star$	`[1, 10, 40]`	0.1
Constant term	1	-	-3

For default parameterization we use $a^\star = 3*2, d^\star = 0.3*2, v^\star = 5*2$. In addition, if the segmentation is used for oculomotor event identification, we add a value of 0.1° to the estimated noise level in order to treat micro-events (eg. microsaccades and tremors) as noise. This value should be increased to match the level of correlated high-frequency noise if such is present due to eg. pre-filtering.

Supplementary figures

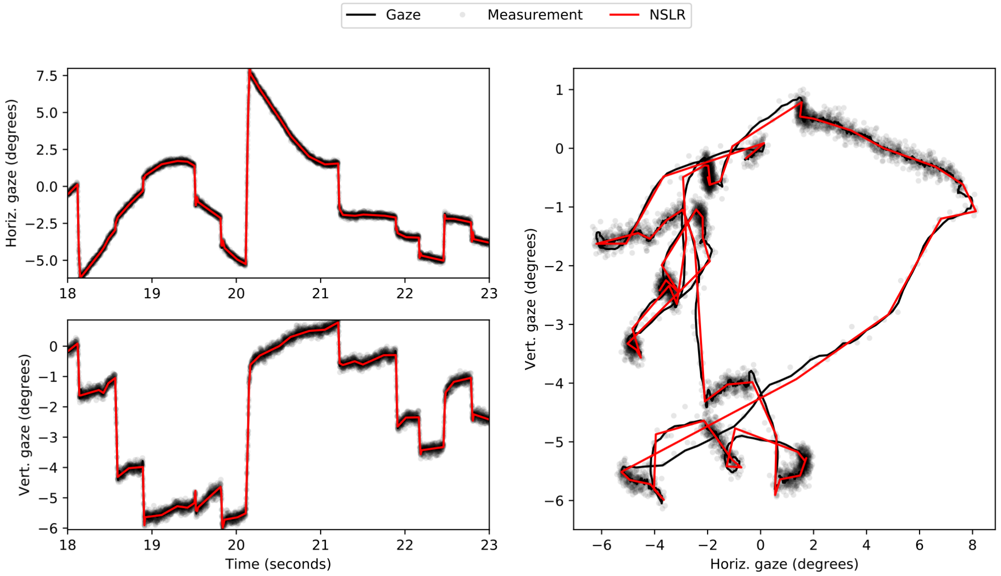

Supplementary Figure S1. Sample denoising result of NSLR with the default parameters and automatically inferred noise level. Recording of a participant watching a movie clip, data from[4]. Simulated noise standard deviation 0.1° per axis.

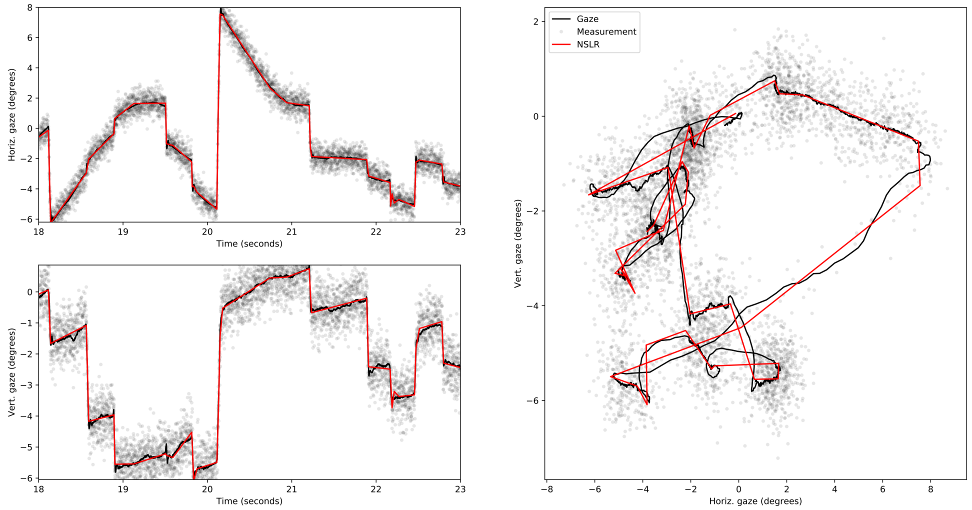

Supplementary Figure S2. Sample denoising result of NSLR with the default parameters and automatically inferred noise level. Recording of a participant watching a movie clip, data from[4]. Simulated noise standard deviation 0.5° per axis.

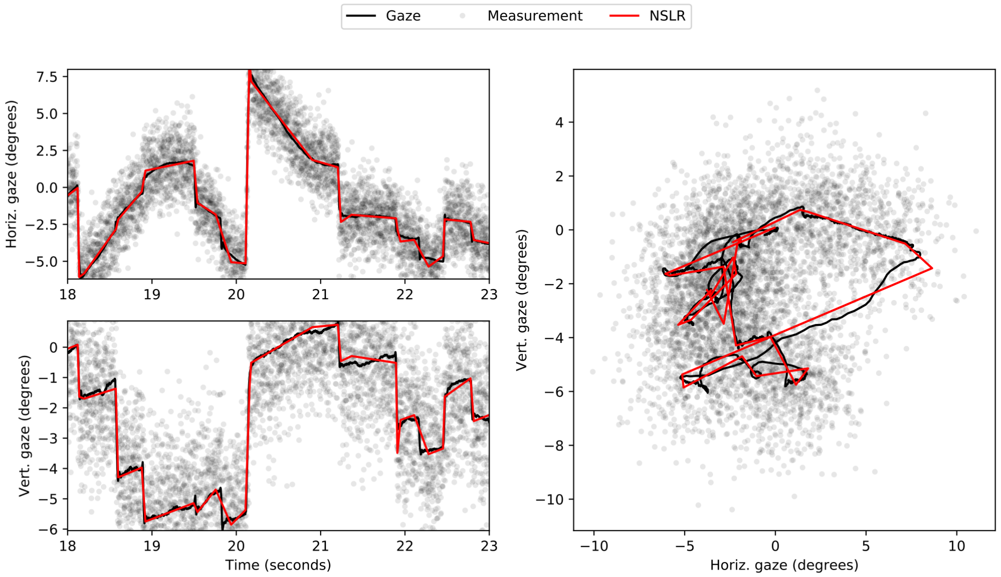

Supplementary Figure S3. Sample denoising result of NSLR with the default parameters and automatically inferred noise level. Recording of a participant watching a movie clip, data from[4]. Simulated noise standard deviation 1.5° per axis.

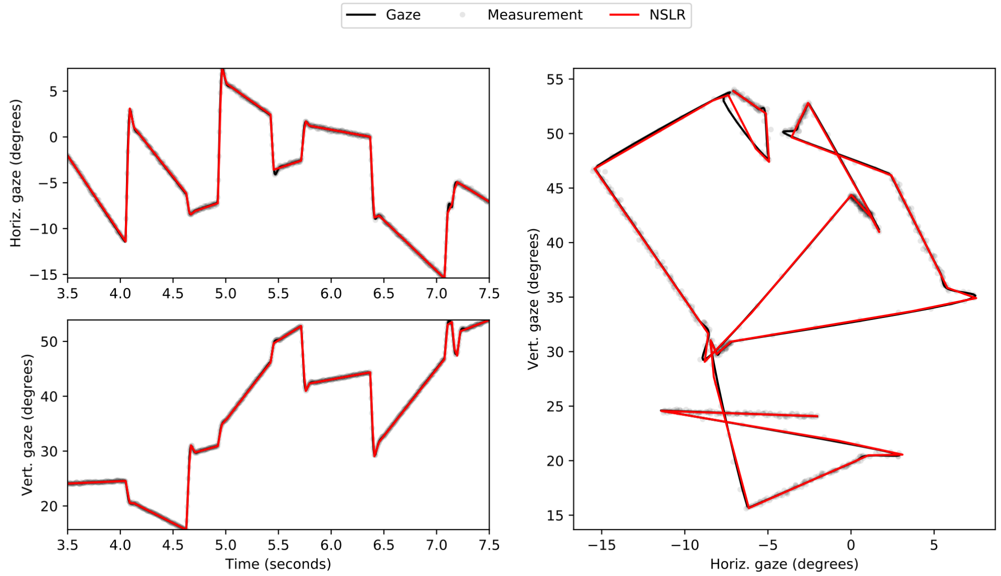

Supplementary Figure S4. Sample denoising result of NSLR with the default parameters and automatically inferred noise level. Simulated data, solid black line indicates simulated gaze signal. Simulated noise standard deviation 0.1° per axis.

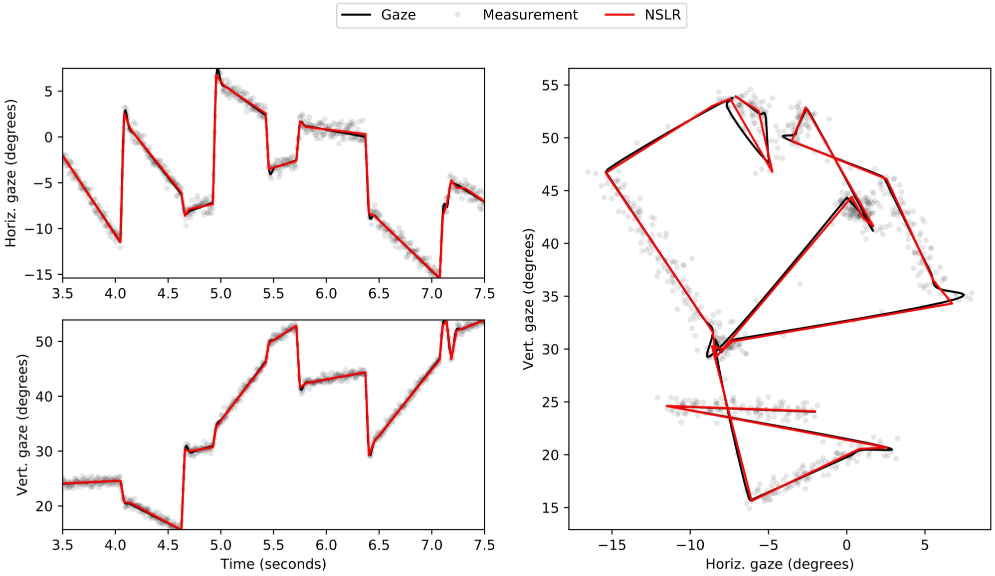

Supplementary Figure S5. Sample denoising result of NSLR with the default parameters and automatically inferred noise level. Simulated data, solid black line indicates simulated gaze signal. Simulated noise standard deviation 0.5° per axis.

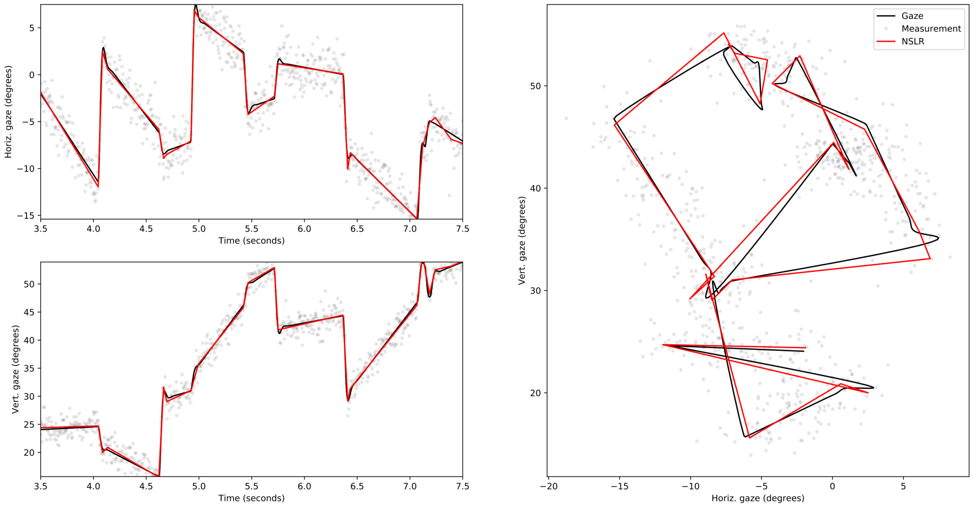

Supplementary Figure S6. Sample denoising result of NSLR with the default parameters and automatically inferred noise level. Simulated data, solid black line indicates simulated gaze signal. Simulated noise standard deviation 1.5° per axis.

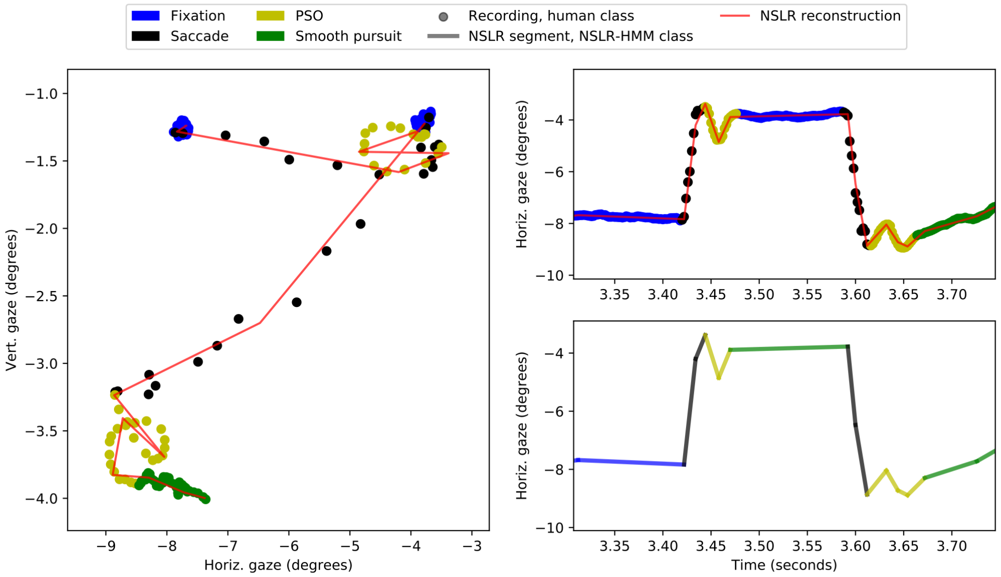

Supplementary Figure S7. Zoomed in classification timeseries for more detail. The segment endpoints match changes in human labeling quite closely. On classification, NSLR-HMM disagrees in this case on the slow segment in the middle, which the human coder classified as a fixation while NSLR classifies it as a smooth pursuit. Data and human labeling from[5].

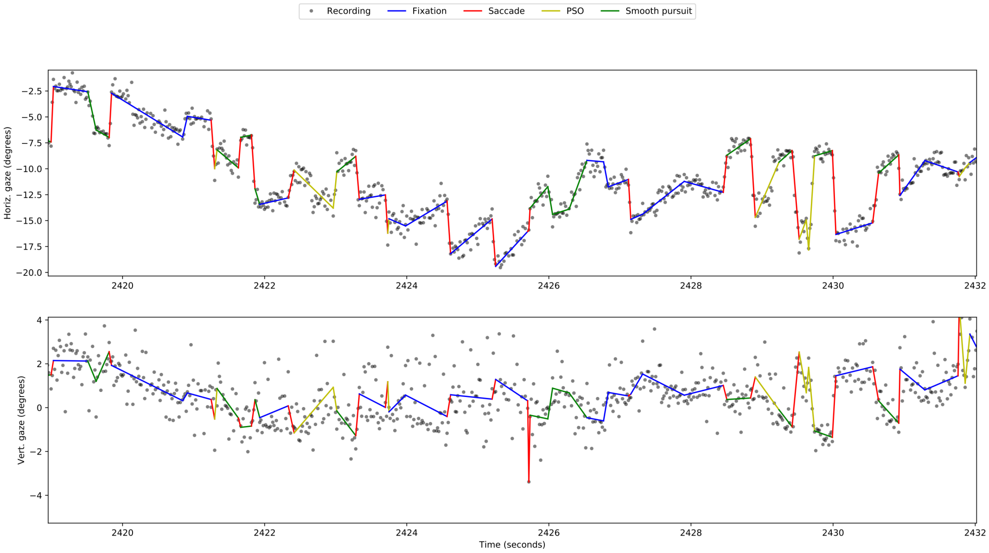

Supplementary Figure S8. A sample NSLR-HMM segmentation and classification with the default parameterization for real-world gaze-in-vehicle data of a participant driving a car. Recorded with a dashboard mounted remote eye tracking system (Smart Eye Pro 5.5) at 60 Hz[6]. The segmentation and classification are in general quite reasonable, although fixations and pursuit eye-movements are hard to tell apart, as the eye is constantly in motion and some spurious detection of post-saccadic oscillations can be seen, especially due to outliers (eg. at about time 2422.5 s).

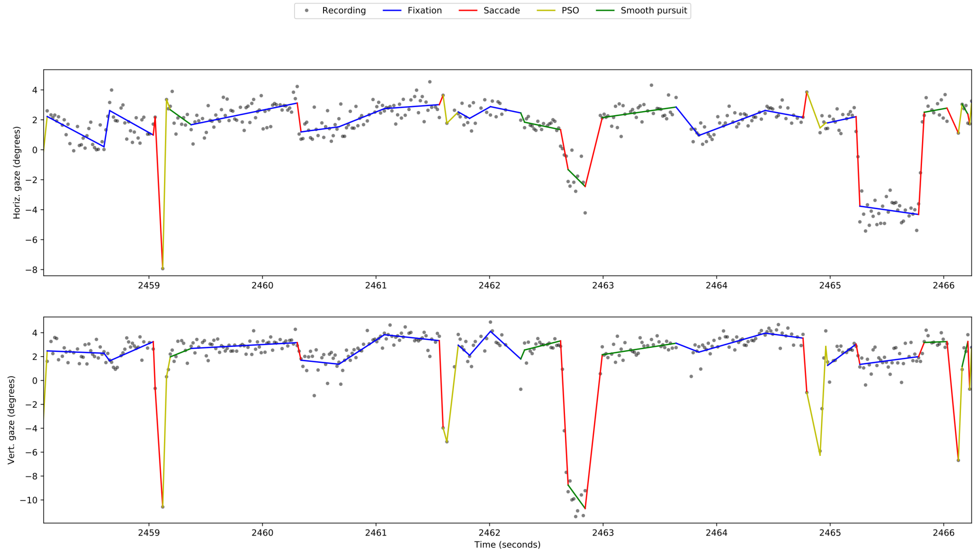

Supplementary Figure S9. A sample NSLR-HMM segmentation and classification with the default parameterization for real-world gaze-in-vehicle data of a participant driving a car. Recorded with a dashboard mounted remote eye tracking system (Smart Eye Pro 5.5) at 60 Hz[6]. The segmentation and classification are in general quite reasonable, although fixations and pursuit eye-movements are hard to tell apart, as the eye is constantly in motion and some spurious detection of saccades post-saccadic oscillations can be seen, especially due to outliers (eg. at about time 2465 s).

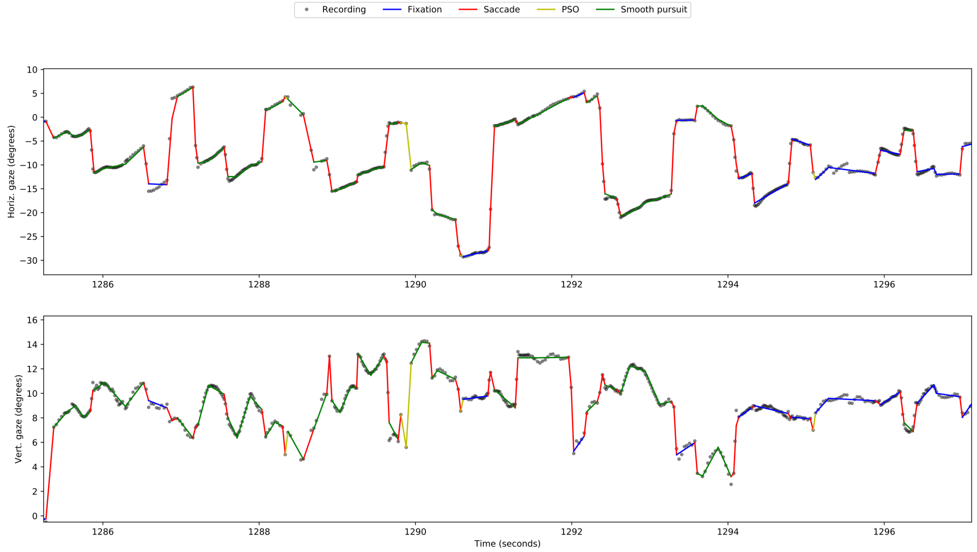

Supplementary Figure S10. A sample NSLR-HMM segmentation and classification with the default parameterization for real-world head-in-eye data of a participant driving a car. Recorded with a head mounted eye tracking system (Pupil Labs Binocular 120) at 30 Hz[7]. The segmentation and classification are in general quite reasonable, although fixations and pursuit eye-movements are hard to tell apart, as the eye is constantly in motion. Some spurious saccades and post-saccadic oscillations are detected due to outliers (eg. at about 1290 s).

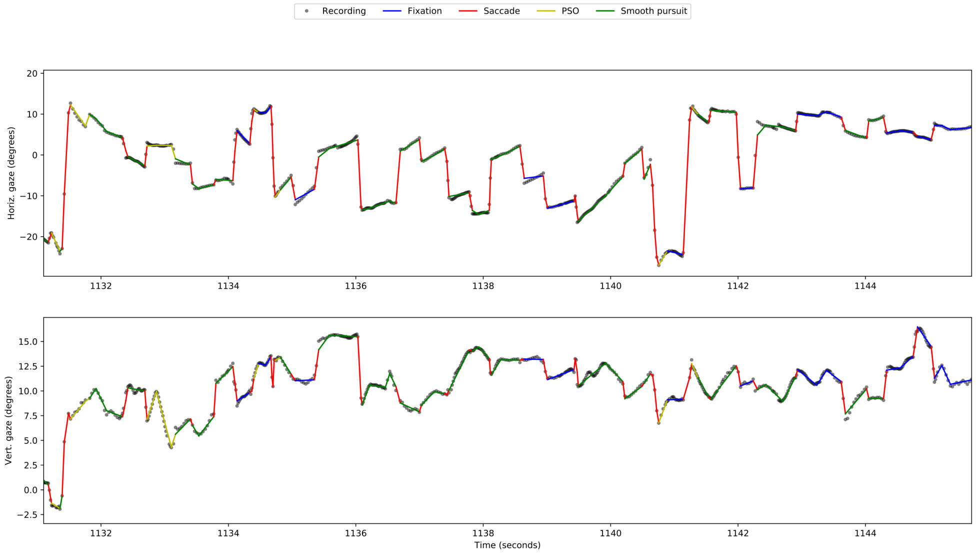

Supplementary Figure S11. A sample NSLR-HMM segmentation and classification with the default parameterization for real-world head-in-eye data of a participant driving a car. Recorded with a head mounted eye tracking system (Pupil Labs Binocular 120) at 30 Hz[7]. The segmentation and classification are in general quite reasonable, although fixations and pursuit eye-movements are hard to tell apart, as the eye is constantly in motion. A relatively fast smooth pursuit, probably due to head motion, can be seen misclassified as a post-saccadic oscillation (at about 1133 s).

References

1. Jackson, B. *et al.* An algorithm for optimal partitioning of data on an interval. *IEEE Signal Process. Lett.* **12**, 105–108 (2005). URL https://doi.org/10.1109%2Flsp.2001.838216. DOI 10.1109/lsp.2001.838216.
2. Killick, R., Fearnhead, P. & Eckley, I. A. Optimal detection of changepoints with a linear computational cost. *J. Am. Stat. Assoc.* **107**, 1590–1598 (2012). URL https://doi.org/10.1080%2F01621459.2012.737745. DOI 10.1080/01621459.2012.737745.
3. Seabold, J. & Perktold, J. Statsmodels: Econometric and statistical modeling with python. In *Proceedings of the 9th Python in Science Conference* (2010).
4. Vig, E., Dorr, M. & Cox, D. Space-variant descriptor sampling for action recognition based on saliency and eye movements. *Comput. Vision–ECCV 2012* 84–97 (2012).
5. Andersson, R., Larsson, L., Holmqvist, K., Stridh, M. & Nyström, M. One algorithm to rule them all? An evaluation and discussion of ten eye movement event-detection algorithms. *Behav. Res. Methods* **49**, 616–637 (2016). URL https://doi.org/10.3758%2Fs13428-016-0738-9. DOI 10.3758/s13428-016-0738-9.
6. Lehtonen, E., Lappi, O., Koirikivi, I. & Summala, H. Effect of driving experience on anticipatory look-ahead fixations in real curve driving. *Accid. Analysis & Prev.* **70**, 195–208 (2014).
7. Lappi, O., Rinkkala, P. & Pekkanen, J. Systematic observation of an expert driver's gaze strategy—an on-road case study. *Front. Psychol.* **8** (2017).
